# Novel Copper (II) Complexes with Fluorine-Containing Reduced Schiff Base Ligands Showing Marked Cytotoxicity in the HepG2 Cancer Cell Line

**DOI:** 10.3390/ijms25179166

**Published:** 2024-08-23

**Authors:** Bianka Oboňová, Jindra Valentová, Miroslava Litecká, Ľudmila Pašková, Jana Hricovíniová, Andrea Bilková, František Bilka, Branislav Horváth, Ladislav Habala

**Affiliations:** 1 Department Chemical Theory of Drugs, Faculty of Pharmacy, Comenius University in Bratislava, Odbojárov 10, 832 32 Bratislava, Slovakia; 2 Department of Materials Chemistry, Institute of Inorganic Chemistry of the CAS, Husinec-Řež č.p. 1001, 250 68 Řež, Czech Republic; 3 Department of Cell and Molecular Biology of Drugs, Faculty of Pharmacy, Comenius University in Bratislava, Odbojárov 10, 832 32 Bratislava, Slovakia; 4 NMR Laboratory, Faculty of Pharmacy, Comenius University in Bratislava, Odbojárov 10, 832 32 Bratislava, Slovakia

**Keywords:** anticancer activity, antimicrobial activity, BSA binding, copper, cytotoxicity, DNA binding, metal complexes, urease inhibition

## Abstract

Several novel copper (II) complexes of reduced Schiff bases containing fluoride substituents were prepared and structurally characterized by single-crystal X-ray diffraction. The complexes exhibited diverse structures, with the central atom in distorted tetrahedral geometry. The biological effects of the products were evaluated, specifically their cytotoxicity, antimicrobial, and antiurease activities, as well as affinity for albumin (BSA) and DNA (ct-DNA). The complexes showed marked cytotoxic activities in the HepG2 hepatocellular carcinoma cell line, considerably higher than the standard cisplatin. The cytotoxicity depended significantly on the substitution pattern. The best activity was observed in the complex with a trifluoromethyl group in position 4 of the benzene ring—the dichloro[(±)-trans-*N*,*N*′-bis-(4-trifluoromethylbenzyl)-cyclohexane-1,2-diamine]copper (II) complex, whose activity (IC_50_ 28.7 μM) was higher than that of the free ligand and markedly better than the activity of the standard cisplatin (IC_50_ 336.8 μM). The same complex also showed the highest antimicrobial effect in vitro. The affinity of the complexes towards bovine serum albumin (BSA) and calf thymus DNA (ct-DNA) was established as well, indicating only marginal differences between the complexes. In addition, all complexes were shown to be excellent inhibitors of the enzyme urease, with the IC_50_ values in the lower micromolar region.

## 1. Introduction

Schiff bases represent a category of compounds with manifold biological effects, such as anticancer, antimicrobial, antiviral, and anti-inflammatory activities [1]. They also act as versatile ligands in metal complexes [2]. The purposeful modification of their structure by varying the substituents on the amine and/or carbonyl components allows for the directed alteration of their chemical, biological, and pharmacological properties. According to the number and character of substituents, they can be mono- to polydentate. Among the most prevalent are the Schiff bases derived from vicinal diamines, especially from cyclohexane-1,2-diamine.

Despite the utility of Schiff base ligands in the preparation of metal complexes, their use is sometimes limited by the relatively low stability of the imine (azomethine) bond due to the reversible character of its formation. The reduction of the carbon–nitrogen double bond imparts much higher stability to these structures, along with a higher geometrical flexibility of the molecule.

The reported Schiff bases of cyclohexane-1,2-diamine derive mainly from substituted salicylaldehydes and are generally tetradentate, while the bidentate Schiff base ligands and their metal complexes based on benzaldehyde derivatives are much rarer, the latter ligands usually being bidentate and uncharged.

Syntheses and X-ray structures of Cu (II) and Zn (II) complexes with reduced Schiff bases formed from unsubstituted benzaldehyde were reported [3]. The complexes were investigated as potential asymmetric catalysts. Both complexes contained one diamine ligand (L) and two chloride ligands, with the general composition [Cu(L)Cl_2_] and [Zn(L)Cl_2_]. The zinc complex was tetrahedral whereas the copper complex exhibited distorted square planar geometry.

A more recent report described the synthesis of a series of *N*,*N*-dibenzyl-cyclohexane-1,2-diamine derivatives [4]. The substituents included -H, -OCH_3_, -NO_2_, -CH_3_, -C_2_H_5_, -F, -Cl, -Br, -i-Pr, -n-Bu, -t-Bu, and CF_3_, at various positions on the benzene ring. The evaluation of the in vitro antimicrobial activities of the products showed that most of them were active against both Gram-negative and Gram-positive bacteria. The compounds also showed a potent antifungal effect, the best antifungal activities generally correlating with the best antibacterial activities. The presence of the cyclohexane ring was found to be essential for the biological effect. The substitution of the benzene ring had a marked influence on the antimicrobial activity. In almost all cases, there was a correlation between the lipophilicity of the compounds and their activity.

A series of reduced Schiff bases were prepared from (*1R*,*2R*)-cyclohexane-1,2-diamine and several substituted benzaldehydes, the substituents including -F, -CF_3_, -OMe, and -NO_2_ [5]. The ligand derived from unsubstituted benzaldehyde was used in the synthesis of a palladium (II) complex.

A nickel (II) complex with two units of (*R*,*R*)-*N*,*N*′-dibenzylcyclohexane-1,2-diamine and two bromide ligands was prepared and its X-ray crystal structure determined [6]. The complex was octahedral, with the two diamine ligands forming the base of an octahedron and the two bromides occupying apical positions.

In continuation of our research on fluorinated Schiff bases and their metal complexes [7,8], we decided to evaluate the biological activity of copper complexes with reduced Schiff bases containing fluoride substituents. We used the ligands described in our previous report [8] to prepare four novel copper (II) complexes. We selected commercially readily available benzaldehyde derivatives containing fluorine substituents (such as -F or -CF_3_ groups). The type and position of these substituents might influence the electron distribution on the aromatic ring and/or influence the products of the cell metabolism of the substances, potentially leading to different biological activity of the substances. The presence of the copper central atom generally confers marked biological activity to the metal complex, especially antimicrobial activity and cytotoxicity, both readily amenable to in vitro measurements. The cell line used in these studies, HepG2, is considered to be susceptible to higher copper concentrations, with the excessive accumulation of copper leading to a progressive loss of viability [9]. Constant (non-toxic) copper levels in the cell are maintained by a combination of regulated import, sequestration, and enhanced export mechanisms, the main mechanism of cytotoxicity being the free radical-induced oxidative damage [10]. On the other hand, the exposure of HepG2 cells to metals can lead to the development of acquired resistance [11], as is also the case with resistance against cisplatin [12].

At the molecular level, the binding of metal complexes to various biomolecules is of considerable importance in the assessment of potential bioactivity and the establishment of a possible mechanism of action; therefore, we investigated the affinity of the copper complexes towards albumin (bovine serum albumin), as it plays a key role in drug pharmacokinetics and pharmacodynamics [13,14], and towards DNA (calf thymus DNA), since the interaction of metal complexes with DNA is among the main mechanisms inducing their cytotoxicity in cells [15,16,17,18]. Copper is also known to exhibit pronounced inhibitory activity against urease, an enzyme implicated in the pathogenesis of several bacterial diseases [19]; therefore, we tested the in vitro activity of the copper complexes against plant urease from *Canavalia ensiformis*.

## 2. Results and Discussion

### 2.1. Synthesis and Characterization

Four new copper complexes were prepared by the addition of an ethanolic solution of copper (II) chloride to the solutions of the respective reduced Schiff base ligands **L11**–**L14** in ethanol. The preparation and characterization of the ligands used in the synthesis, along with their single-crystal X-ray structures, are presented in a previous report [8]. The prepared copper complexes were characterized by the appropriate analytical methods. The results of the elemental analysis correspond to the expected structures of the complexes.

**Figure 1:**
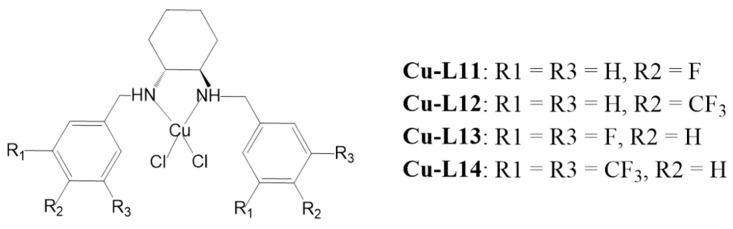
Chemical structures of the **Cu-L11**–**Cu-L14** complexes.

The resulting solid products were then recrystallized from an ethanol/acetonitrile medium and the structure of the obtained blue monocrystals was evaluated by single-crystal X-ray diffractometry. The complexes are poorly soluble in most organic solvents, with the exception of dimethyl sulfoxide (DMSO) and dimethylformamide (DMF). They are stable in DMSO for over 24 h, as determined from the measurement of their UV–Vis spectra in solutions of various concentrations. The complexes are non-ionic, containing two chloride ligands as counter-ions. The chemical structure of the complexes is shown in Figure 1.

The UV–Vis spectra of the ligands are shown in Figures S9–S12 in the Supplementary Materials. The spectra are simple, containing a single absorption band that corresponds to the π–π* transition of the benzene ring. The UV–Vis spectra of the copper (II) complexes (Figures S13–S16 in the Supplementary Materials) exhibit additionally broad charge transfer absorption bands around 300 nm.

The IR spectra provide valuable information about the structure of the copper complexes. In the region above 3000 cm^−1^, symmetric and asymmetric vibrations referring to the two -NH- groups in the molecule can be seen. Their appearance at 3035–3217 cm^−1^ is lower than the expected range of 3400–3300 cm^−1^ that is typical for N-H stretching, which can be indicative of the coordination bond between copper and the electron pair on nitrogen. All four complexes show strong, sharp peaks in the 1100–1350 cm^−1^ region, where C-F stretching bands can be expected. The infrared bands at 2800–300 cm^−1^ can be assigned to stretching bands of the aliphatic C-H from the cyclohexyl group.

### 2.2. X-ray Crystal Structures

**Figure 2:**
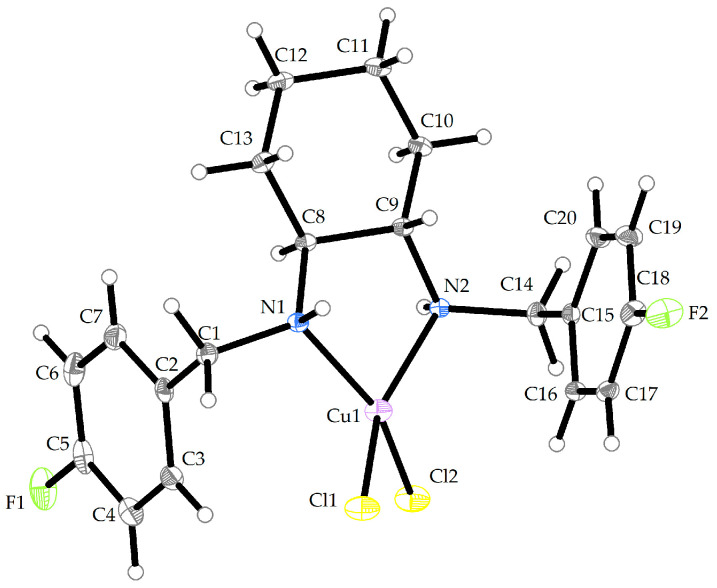
Crystal structure of **Cu-L11** (thermal ellipsoids shown at 30% probability level). The color scheme: gray/white—carbon and hydrogen, yellow—chlorine, green—fluorine, blue—nitrogen, purple—copper.

The compound **Cu-L11** crystallizes in the monoclinic P2_1_/c space group. The molecular structure of the compound **Cu-L11** is shown in Figure 2, and its crystallographic data are summarized in Table S1 (Supplementary Materials). The Cu (II) central atom is surrounded by two chlorido(1-) ligands (Cl1, Cl2) and two nitrogen donor atoms that originate from the organic part of the ligand (**L11**) to form a distorted disphenoid. Selected bond lengths and bond angles are given in Table 1.

**Figure 3:**
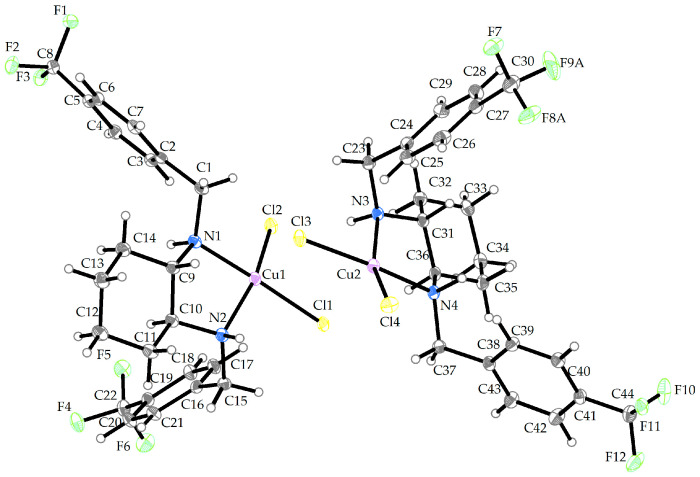
Crystal structure of **Cu-L12** (thermal ellipsoids shown at 30% probability level). Solvent molecules and disordered atoms were omitted for the sake of clarity. The color scheme: gray/white—carbon and hydrogen, yellow—chlorine, green—fluorine, blue—nitrogen, purple—copper.

The compound **Cu-L12** crystallizes in the monoclinic P2_1_/c space group. The molecular structure of the compound **Cu-L12** is shown in Figure 3, and its crystallographic data are summarized in Table S1 (Supplementary Materials). The asymmetric unit consists of two independent molecules with Cu (II) central atoms. Each of the individual Cu (II) central atoms is surrounded by two chlorido (1-) ligands and two nitrogen donor atoms that originate from the organic part of the ligand (**L12**). The coordination polyhedra around the Cu1 (II) and Cu2 (II) central atoms are arranged in a distorted disphenoidal fashion. Selected bond lengths and bond angles are given in Table 2.

**Figure 4:**
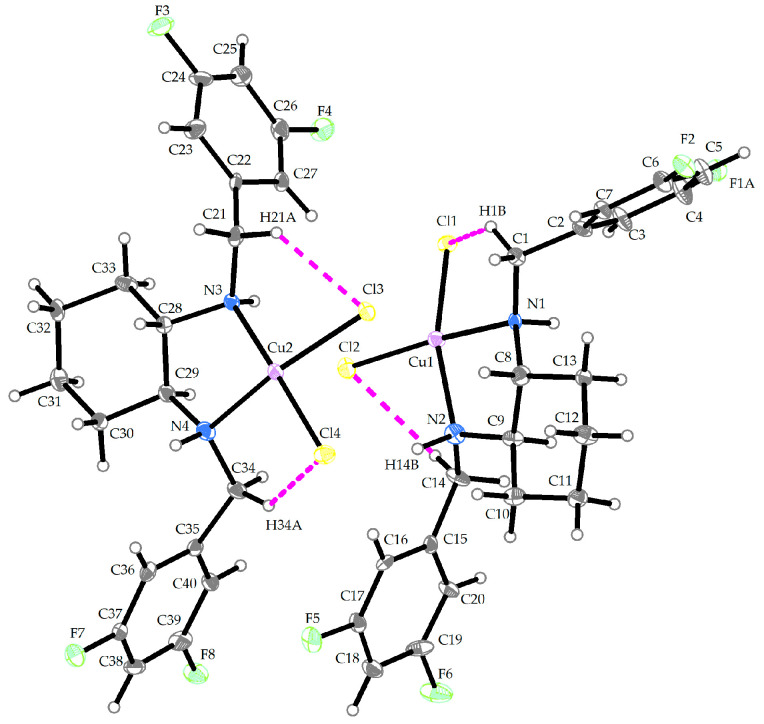
Crystal structure of **Cu-L13** (thermal ellipsoids shown at 30% probability level). The color scheme: gray/white—carbon and hydrogen, yellow—chlorine, green—fluorine, blue—nitrogen, purple—copper.

The compound **Cu-L13** crystallizes in the monoclinic P2_1_/n space group. The molecular structure of the compound **Cu-L13** is shown in Figure 4, and its crystallographic data are summarized in Table S1 (Supplementary Materials). The asymmetric unit consists of the two independent crystallographic units with the Cu (II) central atoms. Each of the Cu (II) central atoms is surrounded by two chlorido (1-) ligands and two nitrogen donor atoms that originate from the organic part of the ligand (**L13**) to form a distorted twisted disphenoid polyhedron. Selected bond lengths and bond angles are given in Table 3.

**Figure 5:**
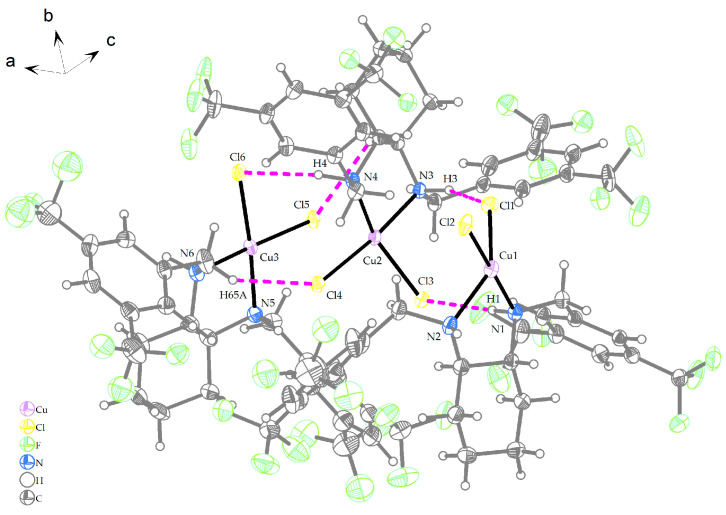
Crystal structure of **Cu-L14** (thermal ellipsoids shown at 30% probability level); organic part was set more transparent. Solvent molecules and disordered atoms were omitted for the sake of clarity. The color scheme: gray/white—carbon and hydrogen, yellow—chlorine, green—fluorine, blue—nitrogen, purple—copper.

The compound **Cu-L14** crystallizes in the monoclinic P2_1_/n space group. The molecular structure of the compound **Cu-L14** is shown in Figure 5, and its crystallographic data are summarized in Table S1 (Supplementary Materials). The asymmetric unit consists of three independent crystallographic units with Cu (II) central atoms. Each of the Cu (II) central atoms is surrounded by two chlorido (1-) ligands and two nitrogen donor atoms that originate from the organic part of the ligand (**L14**). The Cu1 (II) central atom is surrounded by Cl1^−^ and Cl2^−^ ligands and N1 and N2 donor atoms from the organic part to form a distorted heterodisphenoid; the Cu2 (II) central atom forms, with Cl3^−^ and Cl4^−^ ligands and N3 and N4 donor atoms, a distorted twisted disphenoid; the Cu3 (II) central atom is surrounded by Cl5^−^ and Cl6^−^ ligands and N5 and N6 donor atoms in a trapezoid fashion. Selected bond lengths and bond angles are given in Table 4.

### 2.3. Protein Binding Studies

**Figure 6:**
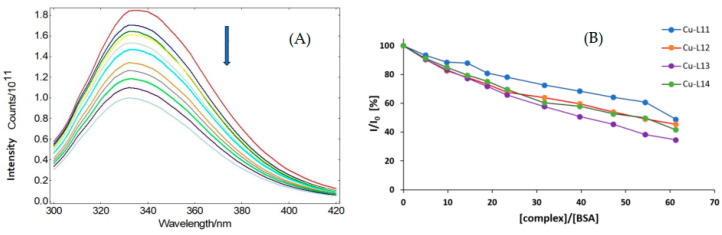
(**A**) Representative fluorescence emission spectra of BSA in buffer solution in the presence of increasing concentrations of the complex **Cu-L11.** The arrow indicates the changes in fluorescence with increasing amounts of the complex. The binding of the copper complexes to albumin causes the induction of a conformational change in BSA and increased quenching of fluorescence in the tryptophane residues present in the molecule of BSA, leading to decreased fluorescence intensity. The concentration of added complexes ranged from 0 to 1.6 × 10^−4^ M (concentration increment 1.6 × 10^−5^ M, indicated by different color of the curve) (**B**) Graphical dependence of relative BSA fluorescence intensity in % at *λ* = 336 nm vs. concentration ratio [complex]/BSA for **Cu-L11**–**Cu-L14**.

Albumin represents the most abundant protein in blood plasma, binding and transporting various kinds of ligands. Interactions between albumin and drugs can significantly influence their pharmacokinetic and pharmacodynamic properties, hence our motivation to investigate the interaction potential of albumin with our complexes. Bovine serum albumin (BSA) was chosen over human serum albumin (HSA) as the model protein target due to the lack of stability of HSA in the citrate and TRIS buffer used in our experiments. Tryptophane residues in BSA exhibit intense fluorescence, with a maximum at 336 nm. After the gradual addition of the tested complexes **Cu-L11**–**Cu-L14** to the BSA solution, we could observe the quenching of fluorescence, as shown in Figures S5–S8 in Supplementary Materials. The plot of relative BSA fluorescence intensity with increasing complex concentration showed a significant quenching of fluorescence, up to 34–61% (Figure 6A). The resultant quenching may indicate the binding of each complex to BSA and the induction of a conformational change in BSA [20].

Dynamic quenching constants (*K_SV_*) and quenching rate constants (*k_q_*) were obtained from the dependence of relative intensity *I*/*I*_0_ on the concentration of the complex (Figure 6B), according to Stern–Volmer equation (Equation (1) in the experimental section). The values of the binding rate constant (*K*) of each complex and the number of binding sites for the complex (*n*) were obtained from the dependency of the relative change in quenching with respect to the concentration of the complex (Δ*I*/*I*_0_)/[complex] on the relative change in quenching Δ*I*/*I*_0_, according to the Scatchard equation (Equation (2) in the experimental section) [21,22]. The values are summarized in Table 5.

The values of the fluorescence quenching rate constant (*k_q_*) of the complexes are between 3.88 × 10^11^ (**Cu-L11**) and 8.46 × 10^11^ M^−1^s^−1^ (**Cu-L13**), which is higher than the threshold value 2 × 10^10^ M^−1^s^−1^, indicating a static quenching mechanism [23]. The values of binding constants (*K*) are in the range of 4.70 (±0.32) × 10^3^ M^−1^ (**Cu-L13**)–8.59 (±0.64) × 10^3^ M^−1^ (**Cu-L12**). These values point to a reversible binding of the complexes to albumin, and the n values for the complexes **Cu-L13** and **Cu-L14** (1.3 and 1.1, respectively) could indicate that there is one independent class of complex binding sites on the albumin. Assuming the fact that there is no significant difference in values between each complex, the position and the type of substituent clearly play only a small role in complex–BSA interaction. The values of the *K* binding constant for the studied complexes are lower than the binding constant (K = 10^−4^ M^−1^) found for a copper (II) complex with a similar Schiff base ligand, *N*,*N*′-bis(3-hydroxysalicylidene)ethylenediamine [24].

### 2.4. Interaction with ct-DNA

**Figure 7:**
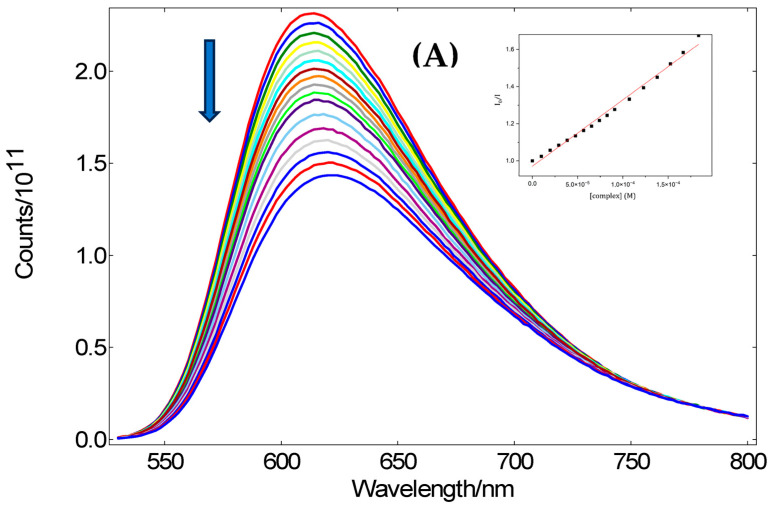

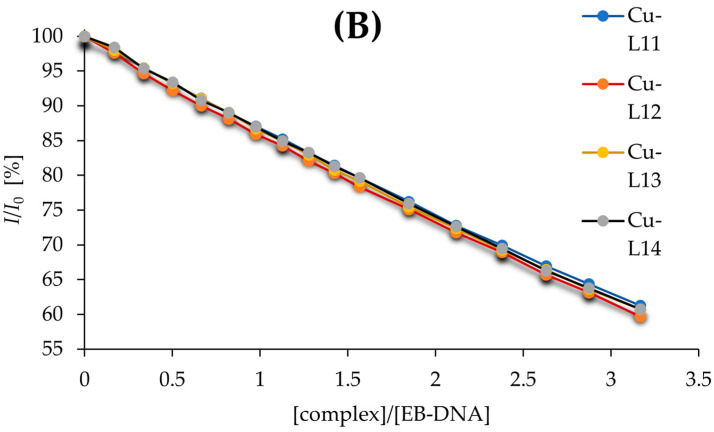
(**A**) Emission fluorescence spectra of EB-DNA in the buffer solution in the presence of increasing amount of complex **Cu-L11**. The arrow indicated the changes in fluorescence at increasing amounts of the complex. Insert graph shows the plot of *I*_0_/*I* vs. [complex]. The concentration of added complexes ranged from 0 to 2 × 10^−4^ M. (concentration increment 2 × 10^−5^ M, indicated by different color of the curve) (**B**) Plot of EB-DNA relative fluorescence intensity (*I*/*I*_0_, % at l = 614 nm vs. concentration ratio [complex]/[EB-DNA] for all tested complexes.

The DNA molecule is one of the key target structures for several anticancer, antibiotic, and antiviral drugs. One of the pathways by which a molecule can interact with DNA and cause replication errors is the intercalation of nucleobases into the DNA strain [16]. The ability of the prepared copper complexes to intercalate into DNA was studied by the displacement of ethidium bromide (EB) from DNA. EB is a typical DNA intercalator and its insertion between adjacent DNA base pairs results in the formation of an EB-DNA adduct that shows an intense fluorescence emission band at 615 nm when excited at 540 nm. The addition of a complex with affinity for DNA lowers the emission intensity of the EB-DNA adduct due to the competition with EB for the same binding sites on DNA. The addition of the tested complexes to EB-DNA quenched the fluorescence, as we can see in Figures S1–S4 in Supplementary Materials. The resultant quenching of the fluorescence reached similar values for all complexes, up to 59–61% of the initial EB-DNA adduct (Figure 7) Values of dynamic quenching constants (*K_SV_*) and quenching rate constants (*k_q_*) calculated according to Stern–Volmer equation (Equation (1) in the experimental section) are summarized in Table 6.

Fluorescence quenching constants values are in the range of 1.48 × 10^11^ M^−1^ s^−1^ (**Cu-L11**)–1.56 × 10^11^ M^−1^ s^−1^ (**Cu-L12**). All emission spectra show a reduction in emission intensity after adding a complex to the EB-DNA solution; however, the reduction of emission intensity is very weak compared to other intercalators [25,26,27]. Similar values for *K_SV_* (1.1–2.9 × 10^3^ M^−1^) were measured for tetradentate Schiff bases with the ethane-1,2-diamine moiety in [28]. The interaction of the complexes with ct-DNA via intercalation might be indirectly suggested. Some studies have shown that non-intercalating DNA groove binding agents are also capable of displacing EB from DNA [29]. The changes in emission and the displacement of EB from the EB-DNA complex following the addition of complexes could be attributed to the competition of the complexes with EB-DNA over binding to the grooves of DNA [30].

### 2.5. Cytotoxicity in Cell Culture

**Figure 8:**
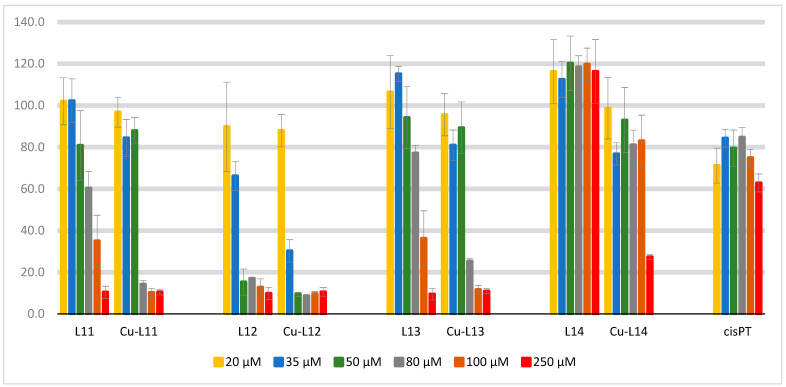
Cell viability (%) of the tested compounds in the concentration range of 20–250 μM. The values for the lowest measured concentrations (2–10 μM) were removed from this depiction for the sake of legibility, as the compounds are generally inefficient at these concentrations.

The cytotoxic activity of the ligands **L11–L14** and their copper complexes against human liver cancer cell line HepG2 was evaluated by the MTT method. Cisplatin was used as a standard compound. The resulting cell viabilities are shown in Figure 8. The values for the lowest measured concentrations (2–10 μM) were removed from the depiction for the sake of legibility, as the compounds are generally inefficient at these concentrations. The calculated IC_50_ values can be found in Table 7.

The ligands **L11–L13** and all copper complexes exhibit concentration-dependent cytotoxicity in the IC_50_ range of 28.7–189.1 μM, with the exception of the ligand **L14,** which shows no activity against HepG2 at any concentration. The highest activity is shown by the copper complex **Cu-L12** with a trifluoromethyl group, with an IC_50_ of 28.7 μM and only 9.40% cell survival at a 50 µM concentration. The activities of the copper complexes are generally higher than their respective ligands. From these results, it can be assumed that complexation of the ligands with the metal ion improves biological activity, i.e., they have a synergistic effect. The coordination of the metal ion with ligands leads to easier transportation through the lipophilic cell membrane, increasing the intracellular accumulation of the metal. Possible mechanisms of cytotoxic activity may involve the interaction of the complex with cancer cell DNA and the well-known ability of redox-active copper ions to generate reactive oxygen species such as O_2_·^−^, OH·, and H_2_O_2_. Interestingly, the cytotoxicity of all the investigated complexes is higher than that of the standard cisplatin, which may be attributed to the intrinsically cisplatin-resistant nature of the currently employed cell line. Due to their genomic instability and complex morphology, hepatocellular carcinoma cells are prone either to be inherently resistant to common chemotherapeutics or to develop resistance within a short timeframe after an initial response [12].

### 2.6. Antimicrobial Activity

Antimicrobial activities of the copper complexes (**Cu-L11**–**Cu-L14**) were evaluated in vitro against Gram-negative (*Escherichia coli*) and Gram-positive (*Staphylococcus aureus*) bacterial strains and against the yeast *Candida albicans*. The activities of representative starting compounds (Schiff base ligand **L1** and reduced Schiff bases **L11 and L12**) were published in [8]. The minimal inhibition concentrations (MICs) of the compounds are shown in Table 8 along with the activity of ciprofloxacine as a standard.

The inhibition activities of the tested complexes against the bacterial strains are much higher than against *C. albicans.* There is no difference in activity against G-positive and G-negative strains, except for the complex **Cu-L13**, where the activity against G-positive *S. aureus* is less potent than against G-negative *E. coli*. The highest activity among the complexes is shown by **Cu-L12**, with an MIC against *S. aureus* and *E. coli* of 0.0267 mM, which is markedly higher than the activities of the remaining complexes. This tendency is already observable in the free ligands, as reported in [8]. The reason for this preference is unclear; it might relate to the production of different metabolites from a particular compound.

We can also observe the increasing antimicrobial activity of the tested compounds after the coordination of the free ligands with the copper ion (e.g., for **Cu-L11**, decrease in MIC against *S. aureus* from 0.7083 to 0.2173 mM). This effect, described in previously published papers [25,31,32,33,34], can be explained based on Overtone’s concept and Tweedy’s chelation theory. According to these theories, lipophilic compounds are more likely to pass through the lipid membrane that surrounds the cell while the chelation of a metal ion is reducing its polarity, increasing the lipophilicity of the compound as well. The antimicrobial activity of copper complexes may be explained by various mechanisms, such as the oxidation of thiol groups, inactivation of enzyme systems, alteration of cell permeability, or inhibition of the respiratory cycle. All of these can prevent the cell from growing and potentially cause cell death [35].

### 2.7. Inhibitory Activity against Urease

The inhibition activities of Schiff base ligands and Schiff base complexes were tested against Jack bean urease isolated from *Canavalia ensiformis* (Jack bean). The results are exhibited as IC_50_ values and are summarized in Table 9. The best activity with an IC_50_ = 2.05 ± 0.34 μM was observed in the **Cu-L14** complex with two trifluoromethyl groups in the phenyl ring. All complexes showed significantly higher activity than acetohydroxamic acid used as a standard (IC_50_ = 185 μM). On the other hand, the free ligands expressed no activity against urease. Copper complexes are known for their good antiurease activity, which can be explained by the strong Lewis acid properties of Cu (II) ions. Some authors suggest copper ions can bind to the thiol groups of histidine residues in the active center of a protein and thus deactivate the enzyme [36,37,38].

## 3. Materials and Methods

### 3.1. Materials and Instruments

All solvents and chemicals were purchased from commercial suppliers (Sigma-Aldrich, Burlington, MA, USA) and were used without further purification. Ligands **L11–L14** were prepared according to the procedure published in the literature [8]. Citrate buffer used for the interaction of complexes with DNA and BSA was prepared by dissolving 15 nM sodium citrate and 150 nM sodium chloride in distilled water. The pH of the buffer was adjusted to 7 using sodium hydroxide or hydrochloric acid. Elemental analyses were carried out using a Flash 2000 CHNS-O Analyser from Thermo Scientific. IR spectra were recorded on a Nicolet 6700 FT-IR spectrometer from Thermo Scientific (Waltham, MA, USA) in the 600–4000 cm^−1^ range. UV–Vis spectra were measured in DMSO with a Genesys 10S UV–Vis spectrophotometer, and the emission spectra were recorded with a FS5 Spectrofluorometer with Standard Cuvette Holder SC-05 (both from Edinburgh Instruments Ltd., Kirkton Campus, Livingston, UK).

### 3.2. Single Crystal X-ray Determination

Data were collected on a Rigaku XtaLAB Synergy S diffractometer equipped with micro-focus CuK*α* radiation and a Hybrid Pixel Array Detector (HyPix-6000HE) (Rigaku, Tokyo, Japan). An Oxford Cryosystems (Cryostream 800) cooling device (Oxford Cryosystems, Oxford, UK) was used for data collection, and the crystals were kept at 100 K during data collection. CrysAlisPro software (version 1.0.43; Rigaku Oxford Diffraction, Yarnton, UK) [39] was used for data collection, cell refinement, and data reduction. Data were corrected for absorption effects using empirical absorption correction (spherical harmonics), implemented in the SCALE3 ABSPACK scaling algorithm, and numerical absorption correction based on Gaussian integration over a multifaceted crystal model. Using Olex2 [40], the structures were solved with the SHELXT [41] (X,Y,Z) structure solution program using Intrinsic Phasing (X,Y,Z) and refined with the SHELXL [42] refinement package using Least Squares minimization. Hydrogen atoms of all molecules were placed in calculated positions. The DIAMOND program (version 2.1e) [43] was used for the molecular graphics. Polyhedral shapes were revised by the Polynator 1.5 software [44]. Cambridge Crystallographic Data Centre (CCDC) contains the supplementary crystallographic data for this article under the CDCC numbers 2361023–2361026. These data can be obtained free of charge via https://www.ccdc.cam.ac.uk/, or from the Cambridge Crystallographic Data Centre, 12 Union Road, CambridgeCB2 1EZ, UK; fax: +44-1223-336-033; or e-mail: deposit@ccdc.cam.ac.uk.

The crystal structures of the ligands (**L11–L14**) along with the corresponding crystallographic methods were described in [8].

### 3.3. Synthesis of the Complexes

To a solution of the ligands **L11–L14** (1 mmol) and sodium acetate (2 mmol) in 50 mL ethanol, a solution of copper (II) chloride (1 mmol) in 20 mL ethanol was added under constant stirring. The mixture was heated to 50 °C and stirred for 3 h, after which it was set aside to cool off to room temperature. After evaporation of the solution, a solid precipitate was formed and filtered. The crude product was washed with distilled water and ethanol, dried in vacuum, and finally recrystallized from ethanol/acetonitrile to obtain blue crystals suitable for X-ray analysis.

Dichloro[(±)-trans-*N*,*N*′-bis-(4-fluorobenzyl)-cyclohexane-1,2-diamine]copper (II) (**Cu-L11**):

Yield: 44%. IR (neat, cm^−1^): 3172 –3120 ν(NH) 2953–2867 ν(CH_2_/CH), 1512, 1222 ν(CF), 843. Elemental anal. for C_20_H_24_Cl_2_CuF_2_N_2_ (464.87) found (calcd. %): N 5.92 (6.05); C 51.95 (51.90); H 4.90 (4.79)

Dichloro[(±)-trans-*N*,*N*′-bis-(4-trifluoromethylbenzyl)-cyclohexane-1,2-diamine]copper (II) (**Cu-L12**):

Yield: 46%. IR (neat, cm^−1^): 3141 ν(NH) 2948–2867 ν(CH_2_/CH), 1621, 1329 ν(CF), 1121, 1069, 852. Elemental anal. for C_22_H_24_Cl_2_CuF_6_N_2_ (564.89) found (calcd %): N 4.71 (4.98); C 45.01 (46.94); H 3.83 (3.94)

Dichloro[(±)-trans-*N*,*N*′-bis-(3,5-difluorobenzyl)-cyclohexane-1,2-diamine]copper (II) (**Cu-L13**):

Yield: 56%. IR (neat, cm^−1^): 3169 ν(NH) 2945–2866 ν(CH_2_/CH), 1378, 1277, 1172, 1128 ν(CF), 885. Elemental anal. for C_20_H_22_Cl_2_CuF_4_N_2_ (500.85) found (calcd %): N 5.43 (5.62); C 47.16 (48.16); H 3.70 (4.04)

Dichloro[(±)-trans-*N*,*N*′-bis-(3,5-bis-trifluoromethylbenzyl)-cyclohexane-1,2-diamine]copper (II) (**Cu-L14**):

Yield: 72%. IR (neat, cm^−1^): 3271 ν(NH) 2937–2860 ν(CH_2_/CH), 1598, 1461, 1313, 1116 ν(CF), 928, 701, 661. Elemental anal. for C_24_H_22_Cl_2_CuF_12_N_2_ (700.88) found (calcd %): N 4.58 (4.01); C 40.50 (41.25); H 2.37 (2.88).

### 3.4. Bioactivity Studies

#### Interaction with Bovine Serum Albumin (BSA)

The interaction of all prepared copper complexes with BSA was studied by quenching the BSA fluorescence. BSA was dissolved in citrate buffer at the initial concentration of 30 µM. The quenching of the emission intensity of BSA tryptophane residues at 336 nm was monitored using the tested complexes (1 mM solution in DMSO) as quenchers with gradually increasing concentrations. The fluorescence emission spectra were recorded in the range of 300–420 nm. The wavelength of the excitation radiation was 280 nm. Measured data were used to study the interaction of a quencher with serum albumins using the Stern–Volmer (Equation (1)) and Scatchard (Equation (2)) equations, obtaining the Stern–Volmer constant *K_SV_* (M^−1^), BSA quenching constant *k_q_* (M^−1^ s^−1^), binding constants *K_BSA_* (M^−1^), and values of the number of binding sites *n* on albumin [45].

(1) $$\frac{I_{0}}{I}=1+k_{q}\tau_{0}\;\left[Q\right]=1+K_{SV}\;\left[Q\right]$$

(2) $$\frac{\Delta I/I_{0}}{\left[Q\right]}=nK_{BSA}-K_{BSA}\frac{\Delta I}{I_{0}}$$

where *I*_0_ = initial tryptophane fluorescence intensity of BSA, *I* = tryptophane fluorescence intensity of BSA after the addition of the complex, *k_q_* = quenching rate constant of BSA, *K_SV_* = dynamic quenching constant, *τ*_0_ = the average lifetime of BSA without the quencher, and [*Q*] = concentration of the quencher. *K_BSA_* is the bovine serum albumin binding constant and *n* is the number of binding sides per albumin.

### 3.5. Quenching of the Fluorescence of EB-DNA Adduct

A stock solution of ct-DNA was prepared by dissolving 6 mg of ct-DNA in 5 mL of citrate buffer. The ability of the compounds to displace EB from its EB-DNA conjugate was investigated by fluorescence emission spectroscopy. The EB-DNA conjugate was prepared by adding 20 µM EB and 26 µM ct-DNA to citrate buffer. The measurements were performed by the gradual addition of the complex solution in DMSO (1 mM) to the EB-DNA conjugate solution and observing the changes in the fluorescence spectra. The spectra were recorded in the range of 550–700 nm and the excitation wavelength was set to 515 nm. The Stern–Volmer constant *K_SV_* was used to evaluate the quenching efficiency of the complexes according to the Stern–Volmer equation (Equation (1)) [46,47].

### 3.6. Urease Inhibition Assay

The urease inhibition activity of complexes was determined using the modified method reported by [48]. The assay mixture, containing 75 μL of Jack bean urease and 75 μL of the tested compounds with various concentrations dissolved in DMSO, was pre-incubated for 15 min on a 96-well assay. Afterwards, 75 μL of phosphate buffer at pH 6.8 (containing phenol red 0.18 mM) and urea (400 mM) were added and incubated at room temperature. We used microplate reader (560 nm) to measure the reaction time required for enough ammonium carbonate to form to raise the pH of the phosphate buffer from 6.8 to 7.7. The end-point of the reaction was determined by the color change of the phenol red indicator. Acetohydroxamic acid was used as a reference.

### 3.7. Cell Growth Inhibition

The cytotoxic activity of the compounds **L11–L14** and **CuL11–CuL14** was evaluated in hepatocellular carcinoma HepG2 cells using the MTT assay. This colorimetric assay measures the reduction of the yellow tetrazolium salt 3-(4,5-dimethylthiazol-2-yl)-2,5-diphenyltetrazolium bromide (MTT) to purple formazan crystals by metabolically active cells [49].

HepG2 cells were seeded in 96-well plates at 70% confluency and pre-cultivated for 24 h before experiment in RPMI 1640 medium (Biosera, Cholet, France) with 10% fetal bovine serum, 1000 U·mL^−1^ penicillin, and 1000 µL·mL^−1^ streptomycin, at 37 °C, 5% CO_2_ (maximum passage number 23).

HepG2 cells were incubated for 24 h with different concentrations (2–250 µM) of the tested compounds. The stock solutions of the compounds were freshly prepared in DMSO (Sigma-Aldrich, Burlington, MA, USA). Cells treated only with the RPMI 1640 medium served as a negative control, medium and DMSO served as a solvent control, and cells treated with cisplatin solution (Accord Healthcare, Harrow, UK) (2–250 µM) served as a positive control. After treatment, HepG2 cells were incubated for 4 h with MTT (Sigma Aldrich) solution (1mg/mL in PBS and complete RPMI medium). Then, the MTT solution was removed, and the formazan crystals were dissolved in DMSO for 30 min. The absorbance intensity was measured using an Epoch Spectrophotometer (BioTek, Winooski, VT, USA) at 570 nm. The experiments were performed in triplicate and cell cytotoxicity was expressed as a percentage relative to the untreated cells. Unexposed cells were used as a control and considered as having 100% cell viability. The viability of cells was calculated using the following formula:

Viable cells (%) = A (treated cells)/A (control cells) × 100

The IC_50_ values were calculated using a four-parameter logistic regression model.

### 3.8. Determination of Antimicrobial Activity

The antimicrobial activities of the synthesized copper (II) complexes were evaluated against *E. coli* CNCTC 377/79 (Gram-negative bacterium), *S. aureus* CNCTC Mau 29/58 (Gram-positive bacterium), and the yeast *C. albicans* CCM 8186. Bacterial strains were obtained from the Czech National Collection of Type Cultures (National Institute of Public Health, Prague, Czech Republic) and the yeast was purchased from the Czech Collection of Microorganisms (Masaryk University, Brno, Czech Republic). Bacteria were grown aerobically in nutrient broth (IMUNA PHARM, Šarišské Michaľany, Slovakia) and the yeast in BD Difco Sabouraud dextrose broth (Becton, Dickinson and Company, Franklin Lakes, NJ, USA) for 18 h at 37 °C or 48 h at 24 °C, respectively. Cultures were then maintained at 4 °C on appropriate solid medium: Endo agar (Oxoid Ltd., Basingstoke, UK) for *E. coli*, blood agar (Biomark Laboratories, Pune, India) for *S. aureus*, and BD Sabouraud dextrose agar (Becton, Dickinson and Company, Franklin Lakes, NJ, USA) for *C. albicans*. Working cultures were prepared by the incubation of a single colony of each microorganism in NB (bacteria) or SDB (yeast) for 18 h at 37 °C or 48 h at 24 °C, respectively. A microbial suspension was prepared in saline solution (0.85% NaCl) according to McFarland standard No 0.5 using a Lambda 35 UV/VIS Spectrophotometer (PerkinElmer Inc., Shelton, CT, USA) to obtain a turbidity (concentration) of ca 1.5 × 10^8^ cfu/cm^3^. After the dilution in the appropriate liquid medium (NB for bacteria and SDB for yeast), a working turbidity of 1.5 × 10^7^ cfu/cm^3^ was prepared.

Antimicrobial activity was determined using the broth dilution method as described in [7]. Stock solutions with initial concentrations of approx. 45 mM for ligands and 27 mM for copper complexes were prepared in DMSO immediately before use. For comparison, ciprofloxacine was used as a standard compound. Working test ligands and copper complexes were prepared by the two-fold serial dilution of stock solutions in sterile doubly concentrated NB or SDB to a final volume 100 mm^3^ within the 96-well microplates. Freshly prepared inoculum (5 mm^3^) of the tested microorganism was added to each appropriate well (bacteria into the plates with NB, *C. albicans* into the plates with SDB). The final turbidity of each microorganism in each well was ca 7.5 × 10^5^ cfu/cm^3^. Working test ligands and copper complexes were prepared by 16 two-fold serial dilutions of stock solutions in sterile doubly concentrated NB or SDB to a final volume 100 mm^3^ within the 96-well microplates. Each concentration was assayed in triplicate. For each test compound and microorganism, the following controls were used: blank, uninoculated media without test compound to account for changes in the media during the experiment; negative control, uninoculated media containing only the test compound; positive control 1, inoculated media without compound; positive control 2, inoculated media with serial dilution of DMSO without test compound, thereby assessing any activity of the solvent. The 96-well plates were incubated aerobically for 24 h at 37 °C or 24 °C, depending on whether bacteria or yeast were grown. Then, 5 mm^3^ of each well was inoculated on the appropriate agar plate (EA for *E. coli*, BA for *S. aureus*, and SDA for *C. albicans*). Bacteria and yeast were grown aerobically for 24 h at 37 °C or 24 °C, respectively. Minimum inhibition concentration (MIC) was defined as the lowest concentration of the compound that inhibited the growth of microorganism on agar plates for all parallel samples compared to the positive controls after 24 h.

## 4. Conclusions

Four copper complexes with reduced Schiff bases were prepared and characterized by X-ray crystallography. Their antimicrobial activity was evaluated against bacterial strains *S. aureus*, *E. coli*, and fungal *C. albicans*, **Cu-L12** having the lowest MIC against both *S. aureus* and *E. coli*. Urease inhibitory activity was tested against Jack bean urease, with all copper complexes showing high activities. Cytotoxic activity was evaluated against the hepatocellular carcinoma HepG2 cell line for reduced Schiff base ligands and their copper complexes. Complex **Cu-L12** with a trifluoromethyl group showed the highest activity, as was also observed in antimicrobial testing. DNA/BSA interaction with copper complexes showed insignificant differences between the complexes; hence, the type of substituent plays only a marginal role in bonding with DNA or BSA structures.

## Figures and Tables

**Table 1:** Selected bond lengths (Å) and bond angles (°) for the complex **Cu-L11**.

Length (Å)		Angles (°)	
Cu1-Cl1	2.2335(4)	Cl1-Cu1-Cl2	100.051(15)
Cu1-Cl2	2.2383(4)	N2-Cu1-Cl1	150.62(4)
Cu1-N2	2.0037(13)	N2-Cu1-Cl2	94.12(4)
Cu1-N1	2.0178(13)	N2-Cu1-N1	85.23(5)
Cu1-Cl1	2.2335(4)	N1-Cu1-Cl1	94.88(4)
		N1-Cu1-Cl2	149.61(4)

**Table 2:** Selected bond lengths (Å) and bond angles (°) for the complex **Cu-L12**.

Length (Å)		Angles (°)	
Cu1-Cl2	2.2813(8)	Cl2-Cu1-Cl1	93.85(3)
Cu1-Cl1	2.3085(8)	N1-Cu1-Cl2	92.22(7)
Cu1-N1	2.039(3)	N1-Cu1-Cl1	173.12(7)
Cu1-N2	2.047(3)	N1-Cu1-N2	84.00(10)
Cu2-Cl3	2.2971(9)	N2-Cu1-Cl2	157.80(7)
Cu2-Cl4	2.2435(9)	N2-Cu1-Cl1	91.55(8)
Cu2-N3	2.038(2)	Cl4-Cu2-Cl3	93.92(3)
Cu2-N4	2.038(3)	N3-Cu2-Cl3	92.43(7)
		N3-Cu2-Cl4	158.17(7)
		N3-Cu2-N4	84.53(10)
		N4-Cu2-Cl3	172.21(7)
		N4-Cu2-Cl4	91.52(7)

**Table 3:** Selected bond lengths (Å) and bond angles (°) for the complex **Cu-L13**.

Length (Å)		Angles (°)	
Cu2-Cl4	2.279(3)	Cl4-Cu2-Cl3	90.10(11)
Cu2-Cl3	2.320(3)	N4-Cu2-Cl4	159.0(3)
Cu2-N4	2.088(9)	N4-Cu2-Cl3	94.1(3)
Cu2-N3	2.057(9)	N3-Cu2-Cl4	91.5(2)
Cu1-Cl4 ^1^	2.618(3)	N3-Cu2-Cl3	176.4(3)
Cu1-Cl1	2.288(3)	N3-Cu2-N4	83.3(4)
Cu1-Cl2	2.317(3)	Cl1-Cu1-Cl4 ^1^	104.85(10)
Cu1-N1	2.039(9)	Cl1-Cu1-Cl2	92.34(11)
Cu1-N2	2.069(9)	Cl2-Cu1-Cl4 ^1^	92.67(11)
		N1-Cu1-Cl4 ^1^	86.2(3)
		N1-Cu1-Cl1	92.4(2)
		N1-Cu1-Cl2	175.3(3)
		N1-Cu1-N2	83.6(4)
		N2-Cu1-Cl4 ^1^	96.6(3)
		N2-Cu1-Cl1	157.9(3)
		N2-Cu1-Cl2	92.0(3)

^1^ -1+X, +Y, +Z.

**Table 4:** Selected bond lengths (Å) and bond angles (°) for the complex **Cu-L14**.

Length (Å)		Angles (°)	
Cu2-Cl4	2.2914(13)	Cl3-Cu2-Cl4	92.18(5)
Cu2-Cl3	2.2567(14)	N3-Cu2-Cl4	173.69(12)
Cu2-N3	2.027(4)	N3-Cu2-Cl3	93.74(12)
Cu2-N4	2.049(4)	N3-Cu2-N4	84.70(17)
Cu3-N6	2.060(4)	N4-Cu2-Cl4	90.96(12)
Cu3-N5	2.040(4)	N4-Cu2-Cl3	153.62(12)
		Cl1-Cu1-Cl2 ^1^	109.13(6)
		Cl2-Cu1-Cl1	92.99(6)
		Cl2-Cu1-Cl2 ^1^	86.54(5)
		N1-Cu1-Cl1	92.07(12)
		N1-Cu1-Cl2 ^1^	90.83(12)
		N1-Cu1-Cl2	174.83(13)
		N1-Cu1-N2	83.93(17)
		N2-Cu1-Cl1	139.27(13)
		N2-Cu1-Cl2	92.89(14)
		N2-Cu1-Cl2 ^1^	111.44(12)
		Cl5-Cu3-Cl6	90.12(5)
		N6-Cu3-Cl5	176.59(13)
		N6-Cu3-Cl6	90.20(13)
		N5-Cu3-Cl5	94.95(12)
		N5-Cu3-Cl6	174.92(13)
		N5-Cu3-N6	84.77(17)

^1^ -X, 1-Y, 1-Z.

**Table 5:** Values of BSA fluorescence dynamic quenching constants (*K_SV_*), BSA fluorescence quenching rate constants (*k_q_*), binding constants (*K*), and number of binding sites per BSA (*n*) of the complexes **Cu-L11**–**Cu-L14**.

Complex	*K_sv_* (M^−1^)	*k_q_* (M^−1^s^−1^)	*K* (M^−1^)	*n*
**Cu-L11**	3.90 (±0.08) × 10^3^	3.88 (±0.08) × 10^11^	5.99 (±0.44) × 10^3^	0.76
**Cu-L12**	5.62 (±0.20) × 10^3^	5.62 (±0.20) × 10^11^	8.59 (±0.64) × 10^3^	0.83
**Cu-L13**	8.46 (±0.26) × 10^3^	8.46 (±0.26) × 10^11^	4.70 (±0.32) × 10^3^	1.37
**Cu-L14**	6.39 (±0.19) × 10^3^	6.39 (±0.19) × 10^11^	5.13 (±0.50) × 10^3^	1.12

**Table 6:** Dynamic quenching constants (*K_SV_*) and EB-DNA fluorescence quenching rate constants (*k_q_*) of the complexes **Cu-L11**–**Cu-L14**.

Complex	Δ*I*/*I*_0_ [%]	*K_sv_* [M^−1^] × 10^3^	*k_q_* [M^−1^ s^−1^] × 10^11^
**Cu-L11**	61.3	3.40 (±0.08)	1.48 (±0.04)
**Cu-L12**	59.7	3.58 (±0.09)	1.56 (±0.04)
**Cu-L13**	60.3	3.51 (±0.08)	1.53 (±0.03)
**Cu-L14**	60.7	3.50 (±0.09)	1.52 (±0.04)

**Table 7:** IC_50_ values for the ligands and their corresponding complexes.

Ligands	IC_50_ ± SD [μM]	Complexes	IC_50_ ± SD [μM]
**L11**	82.1 ± 8.9	**Cu-L11**	61.3 ± 3.4
**L12**	36.7 ± 4.2	**Cu-L12**	28.7 ± 2.0
**L13**	89.1 ± 3.2	**Cu-L13**	64.4 ± 3.2
**L14**	n.a.	**Cu-L14**	189.1 ± 7.1
		cisplatin	336.8 ± 6.7

**Table 8:** Antimicrobial activity MICs (mM) of copper complexes (cpx = ciprofloxacine).

Compound	*S. aureus*	*E. coli*	*C. albicans*
**Cu-L11**	0.2173	0.2173	1.7381
**Cu-L12**	0.0267	0.0267	0.8553
**Cu-L13**	0.4245	0.2123	1.6981
**Cu-L14**	0.2132	0.2132	1.7056
cpx	0.00068	<0.0003	-

**Table 9:** Urease inhibition activities of tested copper complexes.

Compound	IC_50_ ± SD (μM)
ligands	-
**Cu-L11**	2.70 ± 1.09
**Cu-L12**	4.01 ± 0.84
**Cu-L13**	3.47 ± 0.88
**Cu-L14**	2.05 ± 0.34

## Data Availability

The original contributions presented in the study are included in the article/Supplementary Material, further inquiries can be directed to the corresponding author.

## Supplementary Materials

The following supporting information can be downloaded at: https://www.mdpi.com/article/10.3390/ijms25179166/s1.
