# Supplementary material for: Novel Copper (II) Complexes with Fluorine-Containing Reduced Schiff Base Ligands Showing Marked Cytotoxicity in the HepG2 Cancer Cell Line
Source: Int J Mol Sci. 2024 Aug 23;25(17):9166. doi: 10.3390/ijms25179166 (PMC11395566; doi:10.3390/ijms25179166)
Supplement: Supplementary file 1 [file ijms-25-09166-s001.zip › ijms-3152078-supplementary.pdf]

Supplementary materials

Table S1 Crystallographic data for the copper complexes **Cu-L11 – Cu-L14**

Compound	Cu-L11	Cu-L12	Cu-L13	Cu-L14
Empirical formula	$C_{20}H_{24}Cl_2CuF_2N_2$	$C_{45}H_{52}Cl_4Cu_2F_{12}N_4O$	$C_{40}H_{44}Cl_4Cu_2F_8N_4$	$C_{73.6}H_{70.8}Cl_6Cu_3F_{36}N_6O_{0.8}$
Temperature [K]	100(2)	99.99(10)	99.99(11)	100.01(10)
Wavelength [Å]	1.54184	1.54184	1.54184	1.54184
Crystal system	Monoclinic	monoclinic	monoclinic	monoclinic
Space group	$P2_1/c$	$P2_1/c$	$P2_1/n$	$P2_1/n$
Unit cell dimensions [Å]. [°]	a = 12.3334(2)<br>b = 16.1795(2)<br>c = 11.23301(17)<br>α = 90<br>β = 112.5881(18)<br>γ = 90	a = 14.6271(2)<br>b = 9.17461(15)<br>c = 36.7982(7)<br>α = 90<br>β = 97.7699(16)<br>γ = 90	a = 7.3560(2)<br>b = 13.0671(6)<br>c = 42.960(2)<br>α = 90<br>β = 93.353(4)<br>γ = 90	a = 21.3253(3)<br>b = 16.3707(2)<br>c = 26.1742(4)<br>α = 90<br>β = 90.8655(13)<br>γ = 90
Formula weight	464.85	1161.78	1001.67	
Volume [Å^3]	2069.59(6)	4892.92(15)	4122.3(3)	9002.5(2)
Z / Calculated density [Mg/m^3]	4 / 1.492	4 / 1.576	4 / 1.614	4 / 1.579
Absorption coeff. [mm^{-1}]	4.079	3.837	4.289	3.542
F(000)	956.0	23638.0	2040.0	4295.0
Crystal size [mm]	0.14 x 0.05 x 0.04	0.18 × 0.089 × 0.061	0.19 × 0.036 × 0.015	0.39 x 0.11 x 0.06
Theta range for data collection	9.498 - 133.188	6.098 – 133.194°	7.072 - 158.228°	6.846 – 133.194°
Index ranges	-14<=h<=14<br>-19<=k<=19<br>-13<=l<=8	-17<=h<=17<br>-4<=k<=10<br>-43<=l<=43	-8<=h<=8<br>-15<=k<=15<br>-54<=l<=54	-25<=h<=15<br>-19<=k<=19<br>-30<=l<=31
Reflections collected / Independent reflections	22826 / 3651 [R(int) = 0.0329]	8557/ 8557 [R(int) = ?]	10827 / 10827 [R(int) = ?]	54207 / 15768 [R(int) = 0.0307]
Refinement method	Full-matrix least-squares on F^2			
Data / restraints / parameters	3651 / 0 / 244	8557 / 66 / 640	10827 / 12 / 516	15768 / 481 / 1294
Goodness-of-fit on F^2	1.048	1.073	1.033	1.032
Final R indices [I>2σ(I)]	R_1 = 0.0221<br>wR_2 = 0.0565	R_1 = 0.0427<br>wR_2 = 0.1066	R_1 = 0.0878<br>wR_2 = 0.2105	R_1 = 0.0829<br>wR_2 = 0.2263
R indices (all data)	R_1 = 0.0244<br>wR_2 = 0.0577	R_1 = 0.0480<br>wR_2 = 0.1096	R_1 = 0.1519<br>wR_2 = 0.2472	R_1 = 0.0895<br>wR_2 = 0.2332
Flack parameter	n/a	n/a	n/a	n/a
Largest diff. peak and hole [e.Å^{-3}]	0.32 and -0.31	0.79 and -0.55	1.13 and -0.91	1.74 and -0.94

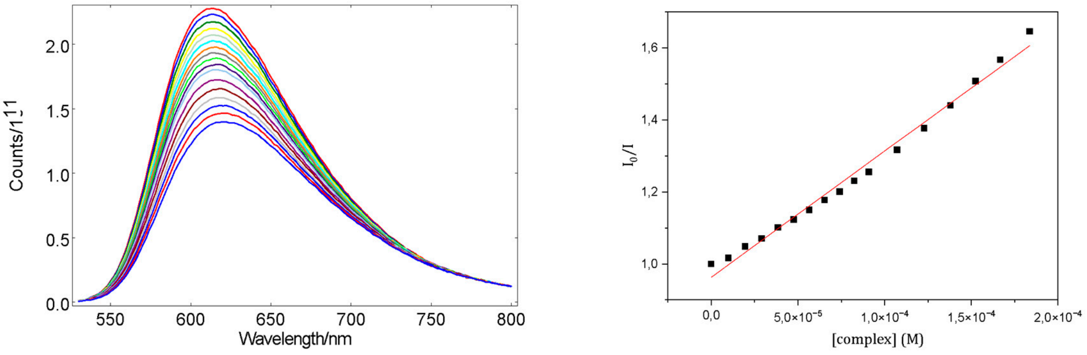

(A) **(B)**

Figure S1 (A) Changes in the fluorescence spectra of EB-DNA upon rising concentration of the complex **Cu-L11**, **(B)** graphical dependence of the relative EB-DNA fluorescence emission intensity (*I/I_0*) vs. concentration ratio [complex]. The concentration of added complexes ranged from 0 to 2 x 10^{-4} M. (concentration increment 2 x 10^{-5} M, indicated by different color of the curve).

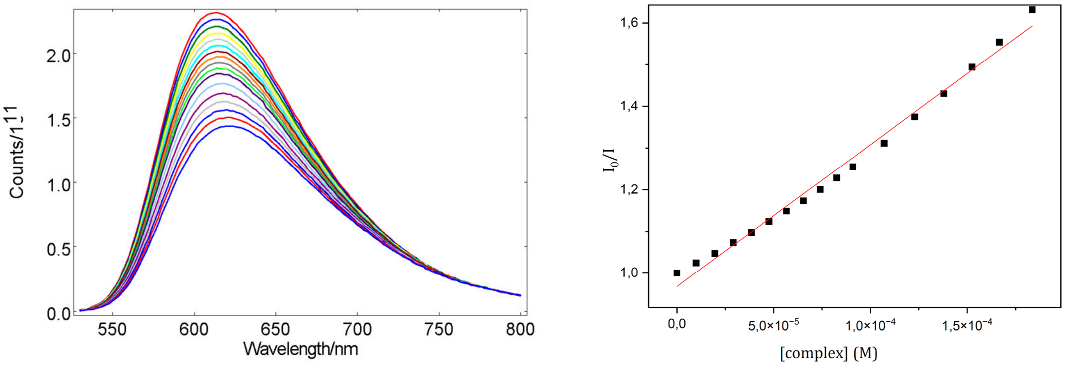

(A) **(B)**

Figure S2 (A) Changes in the fluorescence spectra of EB-DNA upon rising concentration of the complex **Cu-L12**, **(B)** graphical dependence of the relative EB-DNA fluorescence emission intensity (*I/I_0*) vs. concentration ratio [complex]. The concentration of added complexes ranged from 0 to 2 x 10^{-4} M. (concentration increment 2 x 10^{-5} M, indicated by different color of the curve).

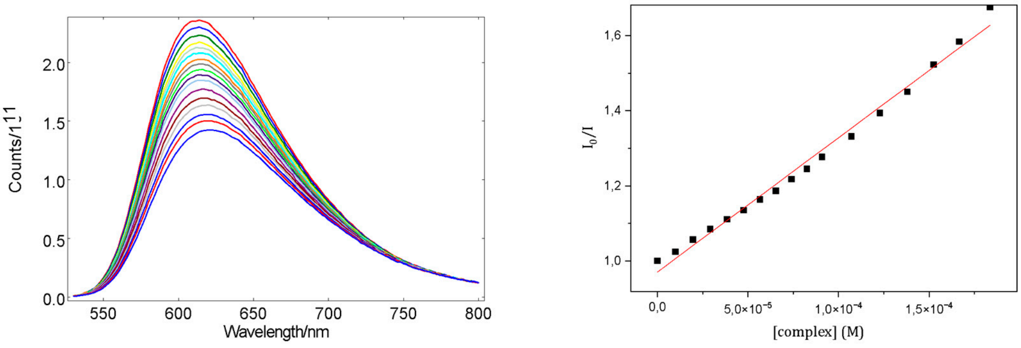

(A) **(B)**

Figure S3 (A) Changes in the fluorescence spectra of EB-DNA upon rising concentration of the complex **Cu-L13**, **(B)** graphical dependence of relative EB-DNA fluorescence emission intensity (*I/I_0*) vs. concentration ratio [complex]. The concentration of added complexes ranged from 0 to 2 x 10^{-4} M. (concentration increment 2 x 10^{-5} M, indicated by different color of the curve).

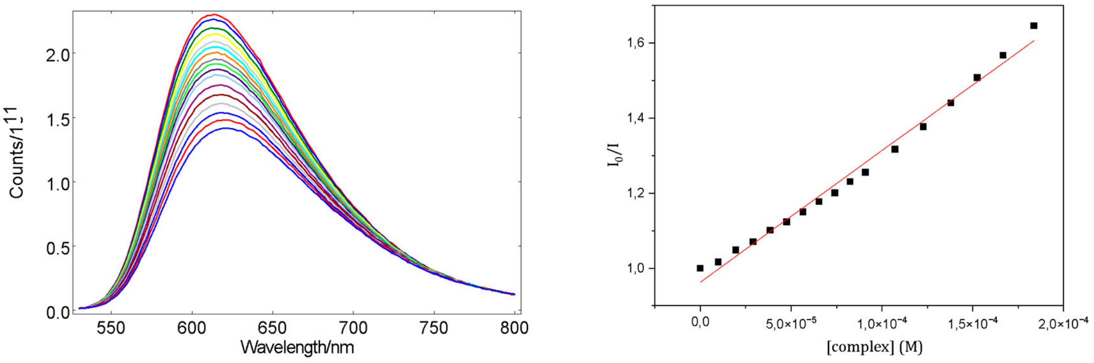

Figure S4 (A) Changes in the fluorescence spectra of EB-DNA upon rising concentration of the complex **Cu-L14**, **(B)** graphical dependence of relative EB-DNA fluorescence emission intensity (*I/I_0*) vs. concentration ratio [complex]. The concentration of added complexes ranged from 0 to 2 x 10^{-4} M. (concentration increment 2 x 10^{-5} M, indicated by different color of the curve).

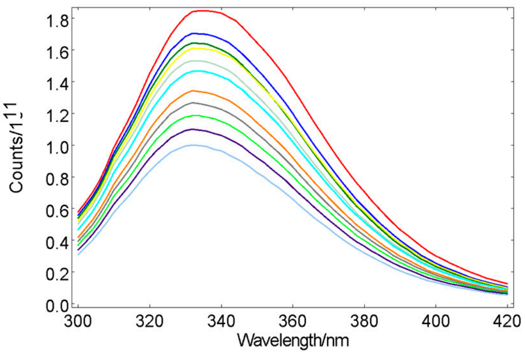

(A)

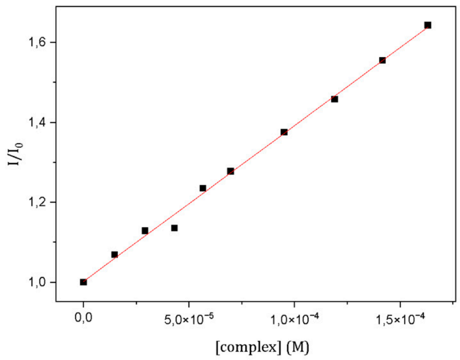

(B)

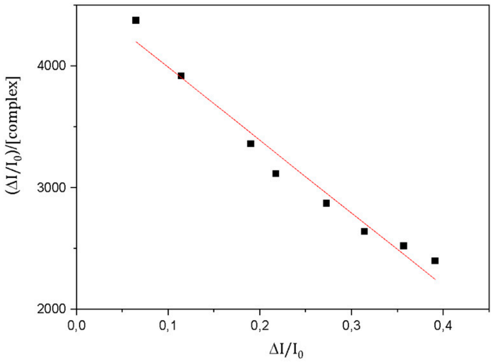

(C)

Figure S5 (A) Changes in the fluorescence spectra of BSA upon rising concentration of the complex **Cu-L11**, **(B)** graphical dependence of relative BSA fluorescence emission intensity (*I/I_0*) vs. concentration ratio [complex], **(C)** graphical dependence of relative changes in BSA fluorescence and concentration of the complex ratio vs. relative changes in BSA fluorescence. The concentration of added complexes ranged from 0 to 1.6 x 10^{-4} M (concentration increment 1.6 x 10^{-5} M, indicated by different color of the curve).

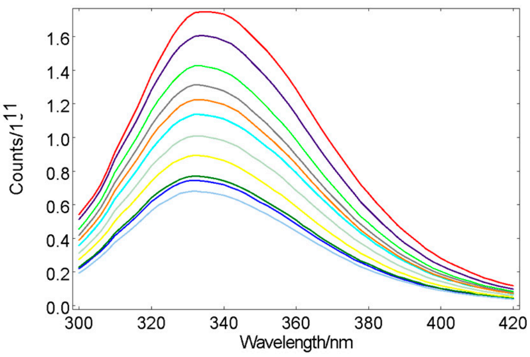

(A)

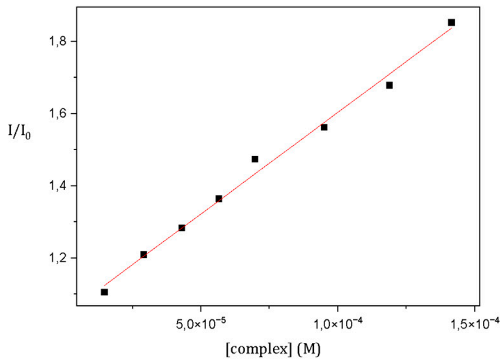

(B)

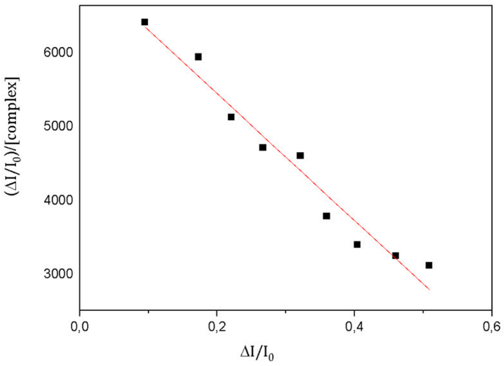

(C)

Figure S6 (A) Changes in the fluorescence spectra of BSA upon rising concentration of the complex **Cu-L12**, **(B)** graphical dependence of relative BSA fluorescence emission intensity (*I/I₀*) vs. concentration ratio [complex], **(C)** graphical dependence of relative changes in BSA fluorescence and concentration of complex ratio vs. relative changes in BSA fluorescence. The concentration of added complexes ranged from 0 to 1.6 x 10^{-4} M (concentration increment 1.6 x 10^{-5} M, indicated by different color of the curve).

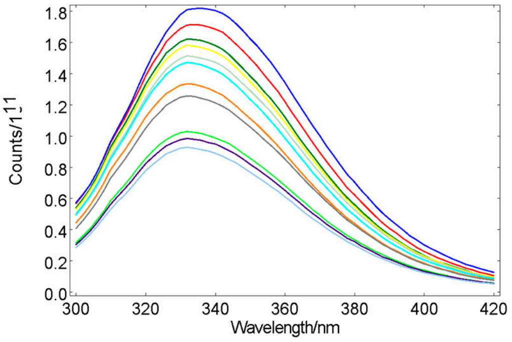

(A)

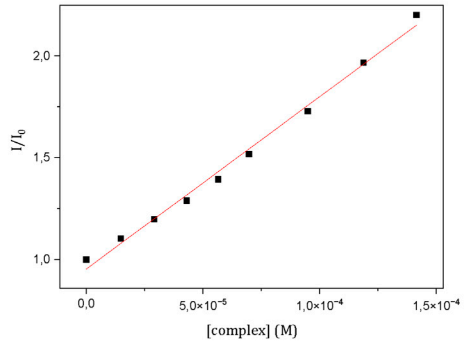

(B)

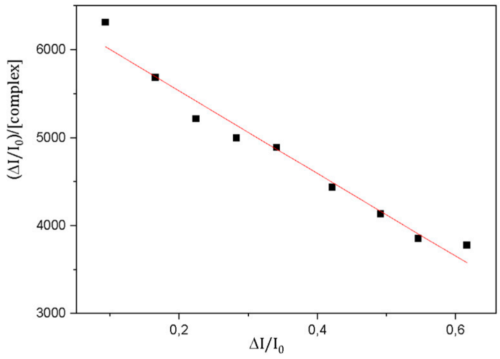

(C)

Figure S7 (A) Changes in the fluorescence spectra of BSA upon rising concentration of the complex **Cu-L13**, **(B)** graphical dependence of relative BSA fluorescence emission intensity (*I/I₀*) vs. concentration ratio [complex], **(C)** graphical dependence of relative changes in BSA fluorescence and concentration of the complex ratio vs. relative changes in BSA fluorescence. The concentration of added complexes ranged from 0 to 1.6 x 10^{-4} M (concentration increment 1.6 x 10^{-5} M, indicated by different color of the curve).

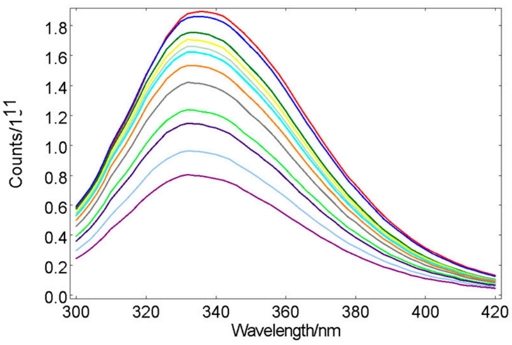

(A)

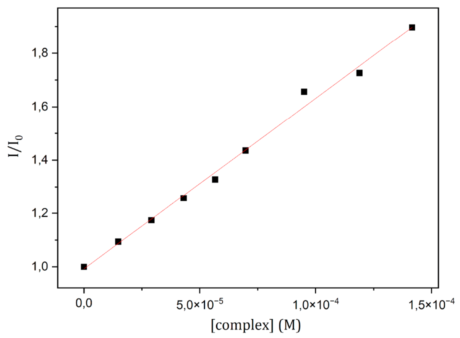

(B)

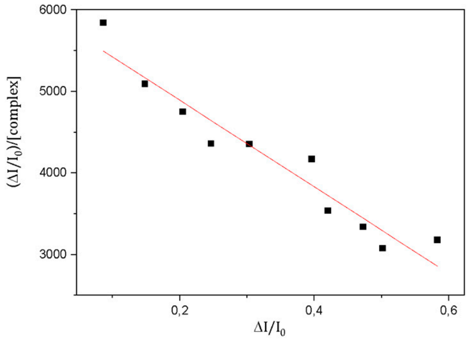

(C)

Figure S8 (A) Changes in the fluorescence spectra of BSA upon rising concentration of the complex **Cu-L14**, **(B)** graphical dependence of relative BSA fluorescence emission intensity (I/I_0) vs. concentration ratio [complex], **(C)** graphical dependence of relative changes in BSA fluorescence and concentration of complex ratio vs. relative changes in BSA fluorescence. The concentration of added complexes ranged from 0 to 1.6 x 10^{-4} M (concentration increment 1.6 x 10^{-5} M, indicated by different color of the curve).

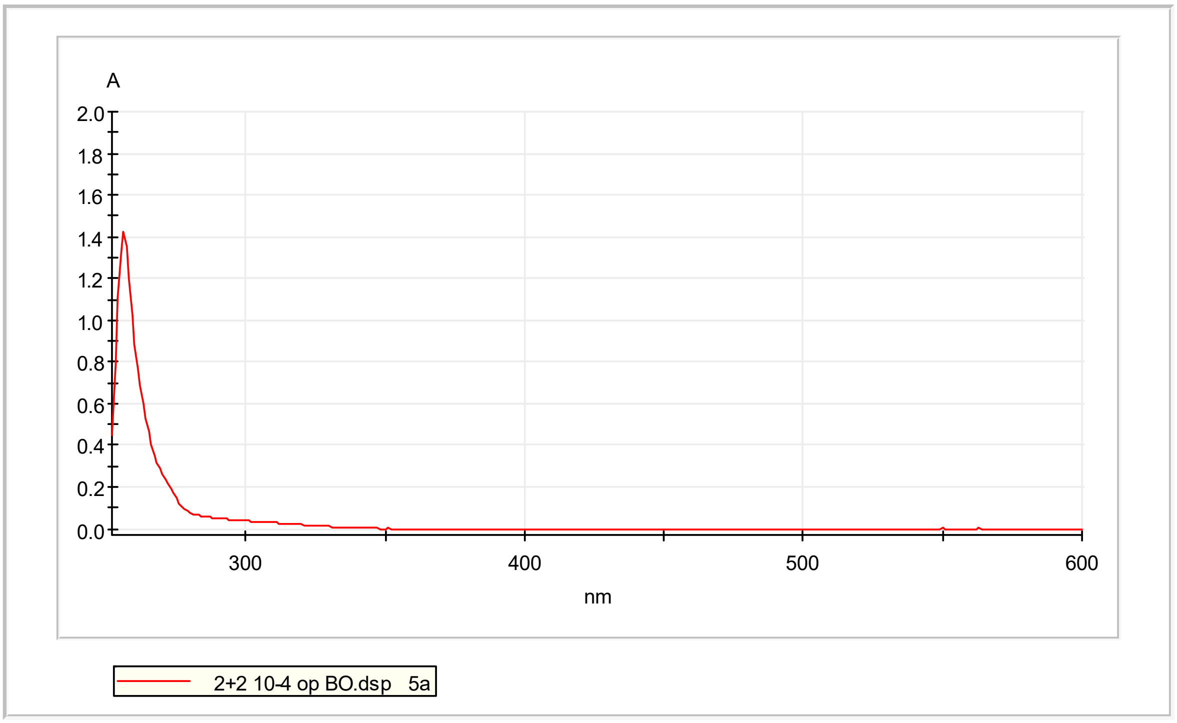

Figure S9 UV-VIS spectrum of the ligand **L11**.

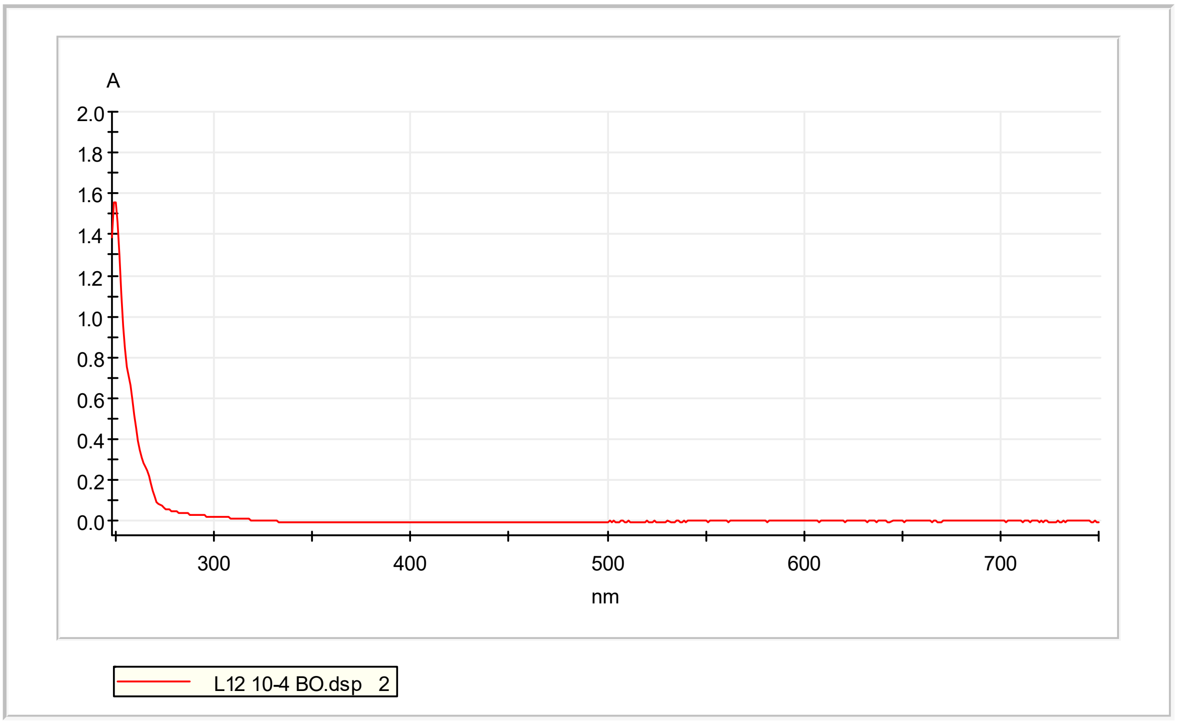

Figure S10 UV-VIS spectrum of the ligand **L12**.

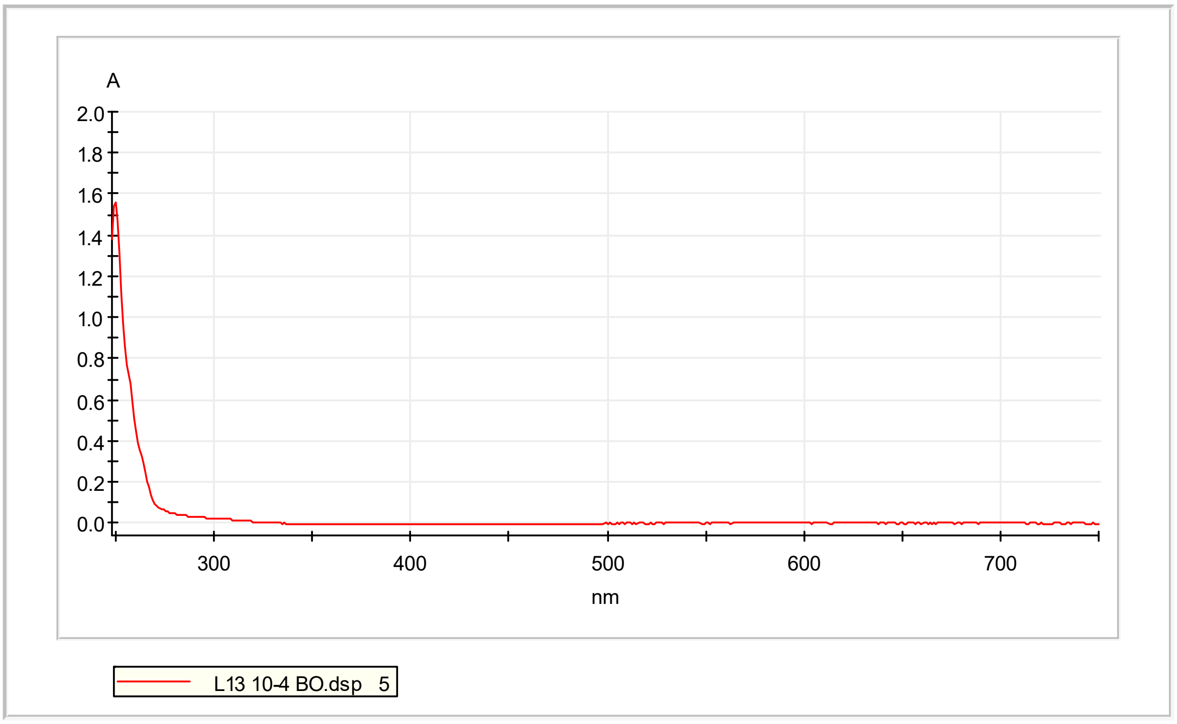

Figure S11 UV-VIS spectrum of the ligand **L13**.

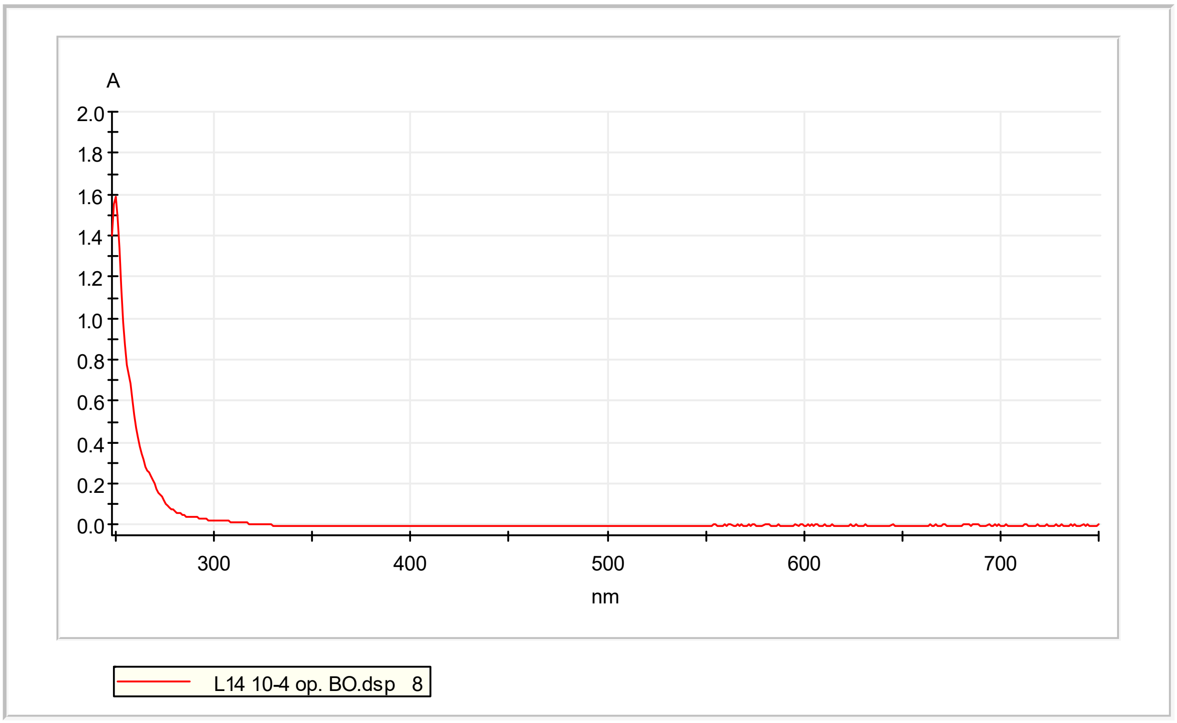

Figure S12 UV-VIS spectrum of the ligand **L14**.

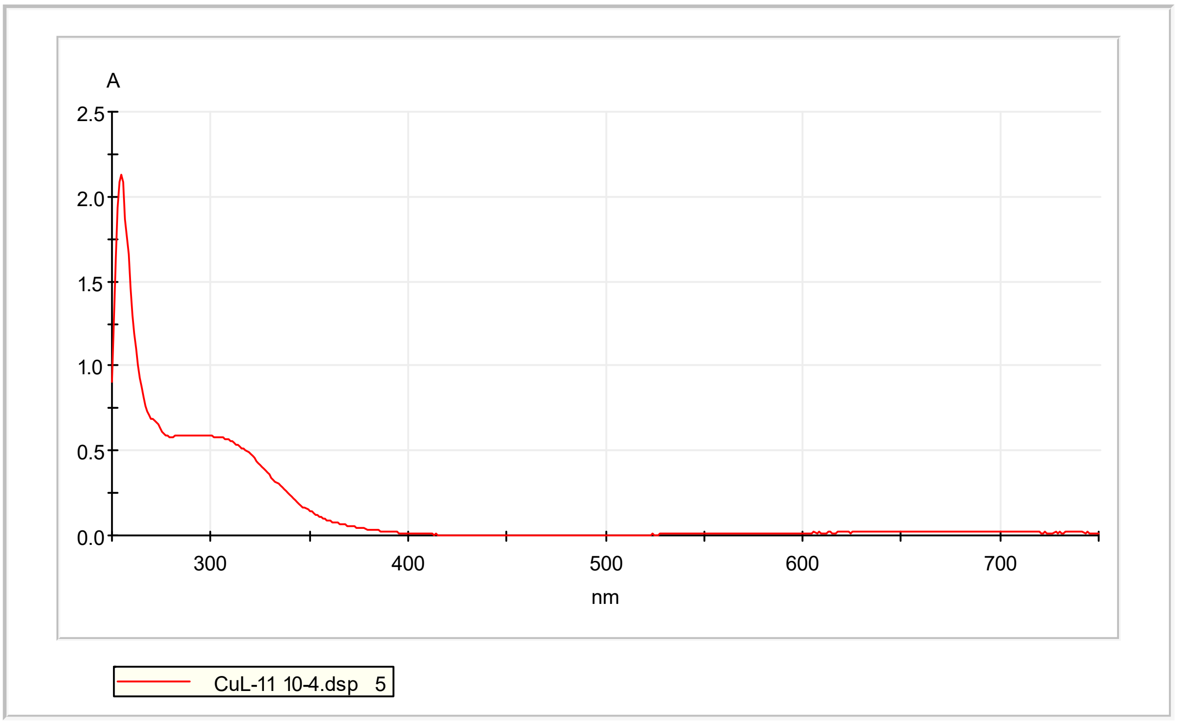

Figure S13 UV-VIS spectrum of the complex **Cu-L11**.

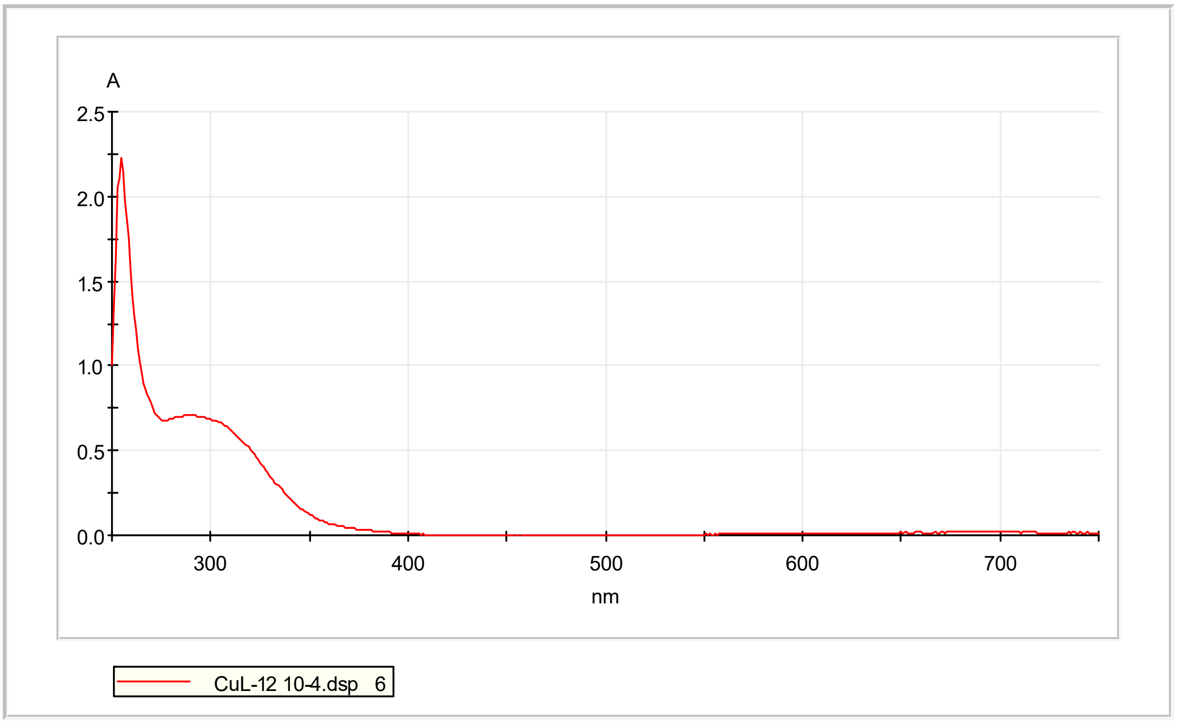

Figure S14 UV-VIS spectrum of the complex **Cu-L12**.

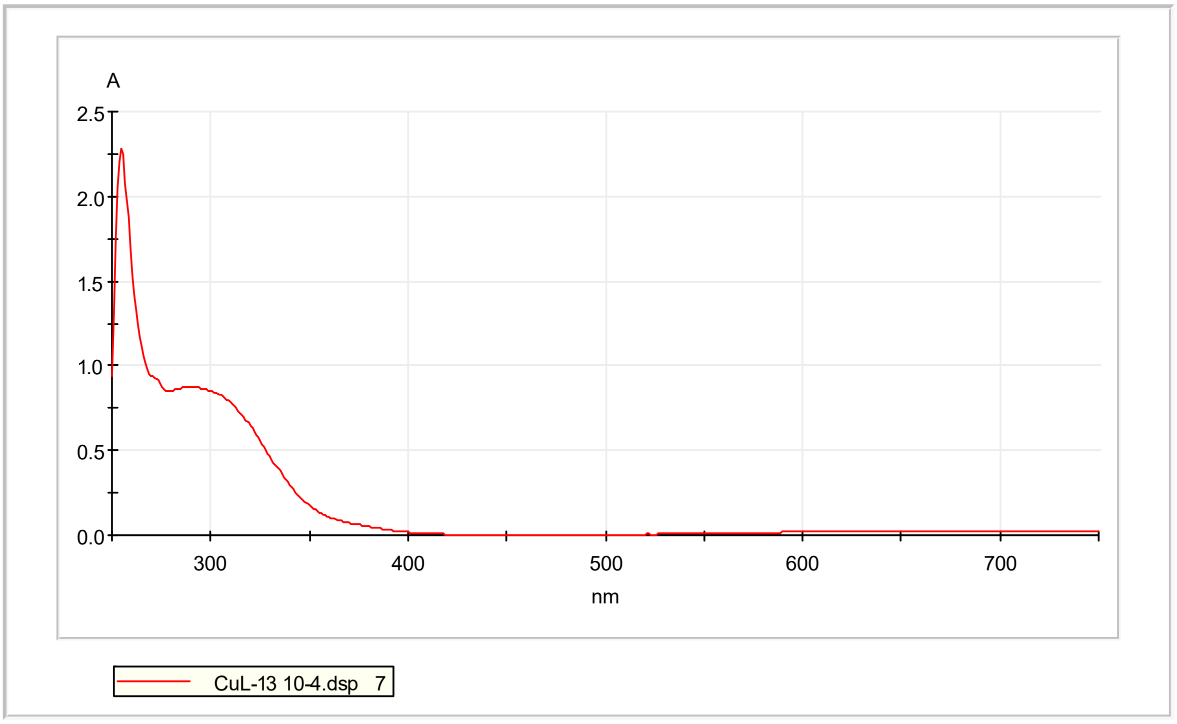

Figure S15 UV-VIS spectrum of the complex **Cu-L13**.

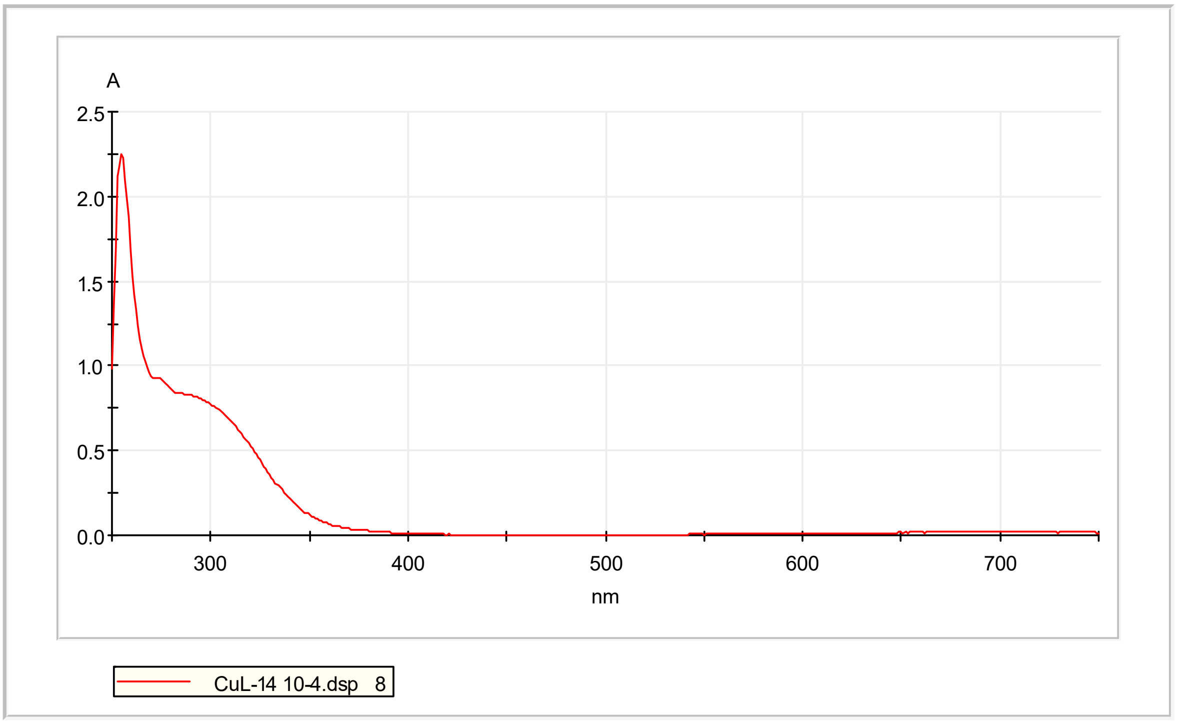

Figure S16 UV-VIS spectrum of the complex **Cu-L14**.
